# South Asian patient experiences of professional interpreting service provision in general practice in England: a qualitative interview study

**DOI:** 10.1186/s12939-025-02477-4

**Published:** 2025-04-16

**Authors:** Graham Hieke, Georgia B. Black, Judith Yargawa, Cecilia Vindrola-Padros, Paramjit Gill, Lily Islam, Emily D. Williams, Sabine Braun, Katriina L. Whitaker

**Affiliations:** 1 School of Health Sciences, University of Surrey, Guildford, UK; 2 Wolfson Institute of Population Health, Queen Mary University of London, London, UK; 3 Department of Targeted Intervention, University College London, London, UK; 4 Warwick Applied Health, Warwick Medical School, University of Warwick, Warwick, UK; 5 Patient and Public Involvement, London, UK; 6 Department of Population Health Sciences, Faculty of Life Sciences and Medicine, King’s College London, London, UK; 7 Centre for Translation Studies, University of Surrey, Guildford, UK

**Keywords:** General practice, Health inequalities, Language barriers, Qualitative methods, Interpreting

## Abstract

**Background:**

Communication difficulties due to unmet language needs are a driver of inequality in healthcare access. The provision of professional interpreting services should mitigate these, and their use is associated with improved patient outcomes. However, interpreting uptake in England is suboptimal and there has been limited research focused on understanding patient experiences and the potential impact on uptake. This multilingual study explored patient perspectives of access to and experience of language support in general practice (primary care) in England, including the use of professional interpreting services and informal language support (i.e. family/friends).

**Method:**

This is a qualitative study based on face-to-face semi-structured interviews with 30 participants from South Asian backgrounds (Pakistani, Indian, Bangladeshi), with no/limited proficiency, living in England. Interviews were analysed with inductive thematic analysis. Patient advisors were involved in all aspects of the research and interpretation of the findings was supported by public engagement focus groups.

**Results:**

Three main themes described participants challenges related to uptake of professional interpreting services including (1) the burden of articulating need, (2) prioritisation of different types of language support (professional/informal), and (3) perceptions of professional interpreting services. Participants described an onus on them to arrange interpreting themselves, whilst regular use of informal language support could inhibit offers of professional language support. Online/digital booking systems perpetuated these challenges. Patient illness appraisal impacted decision making, with informal language support prioritised for less serious matters. Patients highlighted the importance of having confidence in these services, and face-to-face interpreting was preferred to remote interpreting.

**Conclusions:**

No/low English proficiency patients need additional support when booking interpreted-assisted appointments. Increasing patient awareness of and confidence in professional language support is vital to uptake of services. Emphasising the benefits of professional support over informal options is important, with links to patient safety. We provide recommendations for general practice in how to support the uptake of professional interpreting services.

**Supplementary Information:**

The online version contains supplementary material available at 10.1186/s12939-025-02477-4.

## Introduction

The National Healthcare Inequalities Improvement Programme (HiQiP) was established in 2021 to help ensure exceptional healthcare for all, equitable access and improved patient outcomes [1]. Addressing unmet language needs as a barrier to healthcare access and quality is a challenge in primary care in the UK and globally [2]. Limited language proficiency is associated with increased risk of safety incidents [3], including increased risk of physical harm [4]. The use of informal interpreters, including friends or family members, rather than professional interpreters^1^, is also associated with reduced patient safety [5], because of problems such as omitting or altering information [6], as well as raising broader ethical concerns around confidentiality and privacy [7].

General practice is often the first point of access to the healthcare system when people are unwell or experience symptoms. Understanding population need, uptake and resourcing of professional interpreting services is crucial but currently limited [8, 9]. A recent study on the uptake of professional GP-provided interpreting services amongst South Asian populations in England found that more than one-third of participants (37%) with limited/no English language proficiency had not previously used them [10]. Findings from this study indicate that uptake of GP-provided services was more likely amongst those who had been told about the availability of professional interpreting services and those who had been offered a choice of language support, compared with those who had not been told about services/offered a choice. Most had experienced informal or other types of language support, e.g. interpreting by family/friends or bilingual healthcare professionals. Uptake of professional services was less likely amongst those who had previously had friends/family interpret for them during healthcare consultations [10]. However, in-depth qualitative work is required to understand how these factors may influence uptake.

Qualitative evidence to date has used accounts from various relevant parties to understand how consultations change with the introduction of professional interpreters [11], or how users of interpreting services, including staff (GPs, practice nurses, receptionists) adapt services to accommodate language need [12]. Changing migration patterns, alongside increasing complexity in health conditions influenced how interpreting provision was delivered/organised. For example there was increased use of telephone interpreting to provide timely care and access for a broader range of languages [11]. However, there are a lack of studies exploring the perspectives of patients who require the use of language support services in general practice in England, including limited information on the experiences of those who engage with their GP’s professional interpreting services as well as those who chose not to (i.e. use family/friends). Focusing on current patient experiences of professional interpreting services, as well as other types of language support will help identify where improvements may optimise uptake and patient outcomes [13–15].

This study explores South Asian patient experiences of language support in general practice (primary care) in England, including the use of professional interpreting services and informal language support (i.e. family/friends) and to provide a nuanced understanding of potential influences on uptake of professional interpreting services. South Asian communities with limited English proficiency have lower medication adherence [16], and this may interact with increased risk of long-term conditions. For example, South Asians have a greater risk of type 2 diabetes (T2DM) [17], making self-management challenging. Furthermore, South Asian languages combined are the UK’s most commonly spoken languages after English [18].

## Methods

Qualitative face-to-face interviews were undertaken to explore participants’ experiences with professional and informal language support with their primary care provider. This study followed the Consolidated Criteria for Reporting Qualitative Research (COREQ) reporting guidelines [19].

### Participant selection and recruitment

Participants were recruited from respondents (*n* = 609) to a national survey exploring uptake and experience of GP-provided interpreting services [10]. This subsample was selected from survey participants who had agreed (*n* = 372) to participate in a follow-up interview to discuss their experiences of language support at the GP surgery in more detail (*n* = 89 did not agree, *n* = 148 did not respond to this question). Those in the group who agreed to participate in a follow-up interview were more likely to have previously used GP interpreting services (69%) than those who did not (62%). They were also more likely to be from Bangladeshi (40%) and Indian (33%) backgrounds and less likely to be from Pakistani (27%) backgrounds than those who did not agree (Pakistani 52%, Indian 31%, Bangladeshi 17%). Age and sex profiles of the two groups were broadly similar. All interview participants had self-reported limited/no English proficiency, self-identified ethnicity (Pakistani, Indian, Bangladeshi) and had seen or spoken to a healthcare professional from their GP surgery at least once within the last 12 months before survey completion.

### Data collection

A multi-disciplinary team with expertise in interpreting studies, health inequalities and qualitative methods designed two semi-structured interview guides on participant experiences of professional interpreting services and informal language support in general practice (see supplementary materials). A PPI representative also provided input on the development of the interview guides, as well as supporting guidance documents. Open-ended questions explored participants’ most recent experience of a clinical consultation involving either professional or informal language support. This included questions about how language support is arranged; participant decision-making between professional and informal support; the modality (e.g. face-to-face, telephone, video-mediated interpreting) and the role/impact of any technology (e.g. telephone/video-link); and suggestions for future improvements to these services.

Recruitment for interview was informed by quotas to ensure diversity of experiences in the sample, including 20 who had previously used GP interpreting services and 10 who had not done so, across four geographic regions in England (Greater London, Midlands, North West, Yorkshire and the Humber) following purposive sampling criteria (sex, age, and region). Of the eligible participants (*n* = 372), *n* = 60 were approached for interview (a response rate of 50%). All interviews were conducted by trained researchers employed by the sub-contractor responsible for the national survey data collection between May- September 2023 [10]. These researchers, who were fluent in a range of South Asian languages, participated in a briefing session and were provided with guidance on how to conduct interviews. The interviews were conducted in the language of the participants choice. Researchers translated the contents of an information sheet and participants gave verbal consent to participate in the study. A phased start to the data collection enabled detailed feedback to be provided on the initial transcripts, including accuracy checks, and researcher re-briefing where necessary.

### Data analysis

Interviews were digitally recorded and translated/transcribed verbatim into English by a professional translation and transcription agency. Where further clarification was required (e.g. terminology or explanation of meaning), external validation of the transcribed interviews was achieved by an independent multilingual researcher (an independent university employee from a South Asian background). Identifiable information was removed to ensure participant anonymity.

We conducted an inductive thematic analysis supported by MAXQDA 2022 (22.8.0) analytic software. The mean (SD) interview duration was 31 (9) minutes. Our analytic process was guided by a six-phase process [20], incorporating rapid qualitative analysis techniques [21]. All transcripts were repeatedly read by GH to ensure familiarity, whilst four research team members (KLW, GBB, CV, JY) read through all 30 transcripts between them. Open coding conducted by GH resulted in the identification of an initial set of themes. Multiple data analysis meetings took place to refine the themes, involving group analysis of transcripts and production of summaries reflecting the team’s evolving understanding of the themes. Summaries incorporated direct quotations drawn from the data to provide a more in-depth analysis. Theme refinement was also informed by input from a PPI representative [LI] and the wider research team. This reflexive process developed our interpretation and understanding of the findings, ensuring that final themes were representative of the data. We also worked closely with an external organisation, People Street (www.peoplestreet.net), to run three patient engagement focus groups in Bangladeshi and Somali communities within Tower Hamlets (London) to support the interpretation of our findings and reflect on these findings with a group not included as research participants. A total of 30 participants were interviewed. Sample characteristics are reported in Table 1.

**Table 1:** Sample characteristics

Demographics, No. (%)	Full interview sample (*n* = 30)
Sex	
Female	17 (57)
Male	13 (43)
**Age in years: mean (SD)**	49 (12.44)
**Ethnic origin**	
Indian	4 (13)
Pakistani	9 (30)
Bangladeshi	17 (57)
**Main language**	
Bengali/Sylheti	17 (57)
Urdu	9 (30)
Hindi	4 (4)
**English proficiency**	
Not well	10 (33)
Not well at all	20 (67)

## Results

Three main themes describe participants’ experience of using language support to interact with their general practice. These included the burden of articulating need, prioritisation of different types of language support (professional/informal), and perceptions of professional interpreting services. Together these themes reveal several factors that may explain uptake of professional interpreting services in this context.

### The burden of articulating need

This theme explores the challenge of how to articulate need for language support within primary care systems and services; including difficulties using online appointment booking systems (Table 2).

#### Working out the way ‘in’

Participants described their experiences of articulating their need for language support in advance of consultations. Whilst most said they either had to request an interpreter or were routinely provided with one by their GP, others said they had not previously requested an interpreter or their GP practice had not asked whether they needed one, “R [respondent]: *No*,* they don’t do it by themselves*,* I have to tell them.* M [moderator]: You have to tell it yourself, okay. R: *They don’t arrange it on their own.* (528, Sylheti, M, 45). Many participants suggested that this was because of an assumption made by their practice that family members who accompanied participants to their consultations would also act as interpreters, *“[e]very time before an appointment*,* they don’t ask whether bilingual services will be required*,* maybe because they assume that a family member will come along…”* (108, Urdu, F, 36). Just two participants mentioned that their language support need was coded in their medical record, however, this did not mean interpreters were booked automatically, with both relaying situations where an interpreter had not been booked for them.

#### Navigating online booking systems

Language and digital systems presented additional challenges to making the first step to contact general practice for an appointment for a health concern. Even participants who had identified their language need to receptionists were referred to online booking systems. Participants explained that they relied on family and friends to make appointments for them. However, reliance on family members to assist with the booking process appeared to be problematic and presented a barrier to accessing general practice. For instance, some patients were unable to contact their GP surgery during its opening hours as their English-speaking family members were at school/work *“[n]ow appointments and everything other is done online*,* this is hard for me and my husband. Sometimes we get sick but cannot fill up form*,* then the children do it whenever they can make time as they are busy with their work.”* (121, Sylheti, F, 50). The reliance on family members to assist with online appointment requests also inhibited the independent care-seeking behaviour of patients, and it sometimes led to situations where participants had to disclose to those assisting them the reason(s) for seeking a healthcare professional, *“Yes*,* my wife books it. I tell her that I have this problem and she calls the doctor… When she goes in with me*,* she’ll explain to the doctor that I have this or that problem.”* (274, Urdu, M, 29).

**Table 2:** The burden of articulating need

Theme: The burden of articulating need	Example quotations
Working out the way “in”	“…my daughter used to make the appointment, she used to talk about getting Dr. [doctor’s name] appointment. As he was Bengali, I didn’t need an interpreter. But on the previous visit, my daughter told me to do it as I have an appointment and go there. When I went there, I saw that there was an English doctor. Then I said that I don’t understand English, so I need an interpreter. Then they managed through the phone.” (144, Sylheti, F, 60) M: Does the surgery you frequently visit provide information about their interpreter service? R: No. M Okay. R We never inquired about it, nor did they provide any information. We didn’t feel the need since there has always been someone accompanying us, so the need didn’t arise. (190, Urdu, F) “M: So, when you set an appointment for yourself, didn’t they know that your English wasn’t good? R: They knew. That is written in our file. M: Did you ask for an interpreter? R: Yes. Firstly, they didn’t call for an interpreter and when they called for one, I didn’t like the interpreter that much…” (121, Sylheti, F, 50)
Navigating online booking systems	“Now appointments and everything other is done online, this is hard for me and my husband. Sometimes we get sick but cannot fill up form, then the children do it whenever they can make time as they are busy with their work.” (121, Sylheti, F, 50) “M: Don’t you have anyone to help you with the online stuff? R: Yes, I have, but they are busy. And there are many problems which I cannot tell my relatives about. M: Okay. R: I want to talk to the doctor directly. The people at the reception should help me but they don’t, they ask me to get an online appointment.” (528, Sylheti, M, 45) “I don’t call because if I call them, I don’t understand if I have to make an appointment, what do I have to say, sometimes I don’t understand, so my husband calls them and takes an appointment.” (209, Hindi, F, 38)

### Prioritisation of other options of Language support

This theme discusses how other types of language support were prioritised, depending on factors such as perceived benefits of using informal interpreters and interpretation of the presenting symptom/issue (Table 3).

#### Perceived benefits of using family members/friends as interpreters

Many participants mentioned family and friends who were often seen as a better source of language support than professional interpreters due to alignment with dialect, trust and comfort, *“conversations with the interpreters are okay but they have regional dialects. Therefore*,* I take the children with me to the GP surgery instead of using the interpreters. As I can understand them better than the interpreters.”* (8 Sylheti, M, 63). Others acknowledged their reservations about disclosing or discussing certain issues in front of family members, especially children, *“[s]o when it comes to female problems*,* there is no need to involve the child…”* (188, Hindi, F, 46). Several participants mentioned concerns about confidentiality and embarrassment when needing to disclose information about health conditions to family and friends. One participant also cited concerns about the ability of informal interpreters to interpret accurately, *“[t]he informal ones that I used are not good for me*,* there are confidential things which I have to talk about in front of my friends. It’s embarrassing for me; I get the feeling that he will talk about it to someone else. Then there is doubt about whether he can properly explain the problem or not.”* (113 Sylheti, M, 56). GP practices seemed to influence this decision, with some mandating that use of family and friends as interpreters should be avoided, *“they [GP practices] say that a family member can’t come with you because they won’t tell you what will happen*,* what won’t. They will keep some things to themselves because they think you’ll get worried.”* (84, Urdu, F, 35).

#### Symptom/illness appraisal impacts choice of Language support

The presenting symptom/issue also impacted decision-making about language support. For example, participants reported being willing to attempt to communicate without a professional interpreter for problems they perceived to be less serious, *“if there are trivial problems*,* I can understand them myself. But if the problem is too difficult then we must use an interpreter. I need it then.”* (445, Sylheti, F, 39).

**Table 3:** Prioritisation of other options of Language support

Theme: Prioritisation of other options of language support	Example quotations
Perceived benefits of using family/ friends as interpreters	“I liked when my children came with me. If I didn’t understand the whole thing at the GP, they could explain it to me back home.” (139, Sylheti, M, 67) “when I’m with my wife I feel very comfortable because what I want to say, my wife will say it in English. And what the doctor says in English, I know she’ll tell me properly. So I know that the appointment will go well. (274, Urdu, M, 29) “[t]here are certain physical ailments where conveying it through someone from your family becomes very hard, there is an embarrassment. There are many things like that which you cannot say” (8 Sylheti, M, 63).
Symptom/ illness appraisal impacts choice of language support	“Sometimes I take my sister. Sometimes I understand… it depends on what the problem is. If it’s a small matter then I don’t need it [an interpreter]. But if something is more important or at a point where it’s a matter of concern, then interpreter. So, I understand what’s going to happen, what they’ll do. But not every time. Maybe if it’s a small headache then I can talk about it myself.” (84, Urdu, F, 35). “we have mostly gotten an interpreter. However, we don’t get it all the time, if there are trivial problems, I can understand them myself. But if the problem is too difficult then we must use an interpreter. I need it then.” (445, Sylheti, F, 39)

### Perceptions of professional interpreting services

The final theme presents participants’ perceptions of professional interpreting services including the relationship to care and preferences about the type of service offered (Table 4).

#### Perceived variation of service quality and impact on care

Challenges finding interpreters led to appointment delays and was a source of frustration. A limited number of participants also reported challenges during consultations involving interpreters, such as feeling unable to elaborate or express themselves, *“[y]ou cannot talk a lot. I answered what the doctor asked. Then he asked something else and I answered back.”* (139, Sylheti, M, 67), or that interpreters “*cannot convey what we say to the doctor”* (121, Sylheti, F, 50). Whilst there was evidence that quality of the interpreting could vary, several participants spoke about the benefits of using professional interpreting services. These included interpreters helping patients to explain and have their issues understood by staff, as well as alleviating the emotional response to seeking help, *“…[t]hey are making me understand that there is nothing to fear when I got to the doctors…”* (145, Sylheti, F, 35). Some highlighted benefits including privacy and confidentiality, *“when using a professional interpreter*,* the privacy is maintained*,* no one will know*,* I can openly talk to him about my problem and he can explain my confidential issues to the doctor. That is what I think is good.”* (113 Sylheti, M, 56). Participants reported a tension between recognising the benefits of using professional interpreters (e.g. comprehension), *“[t]he advantage is that she [the professional interpreter] can explain everything well about what the problem is*,* what’s going to happen. So*,* the understanding is good…”*, *with concerns about disclosing information about their health to a third party i.e.*, *the interpreter*,* “…[b]ut obviously the problem is with me. I feel that my problem should be known only to me. So I don’t like that someone else knows. Of course*,* it’s confidential*,* that’s their job and we do need them*,* but for us it’s just the fact that it’s someone else.”* (84, Urdu, F, 35).

#### Preference of face-to-face interpreting

Participants demonstrated a preference towards face-to-face interpreting rather than remote (e.g. telephone) solutions; this was largely due to a better ability to understand each other. *“[i]t seems to me that if you talk face-to-face*,* you can understand better. On the telephone*,* it cannot be understood often.”* (359, Sylheti, F, 42). People also described experiencing technical challenges with telephone interpreting, leading to inconsistency (e.g. issues re-connecting the same interpreter). Other participants perceived benefits from the anonymity of telephone interpreting; “*That would be more comfortable because she can’t see you. What if you go shopping and she saw you; she knows everything about you. Someone who’s on the phone can’t see your face.”* (84, Urdu, F, 35).

**Table 4:** Perceptions of professional interpreting services

Theme: Perceptions of professional interpreting services	Example quotations
Perceived variation of service quality and impact on care	“You are not able to communicate, so in the meantime the illness you had always goes away. Because they call you after three or four days, and by that time you would have already done a home remedy, or taken medicine from the pharmacist.” (372, Hindi, F, 73) “Sometimes I get some interpreters who make the GP understand properly. In detail. And there are some who can’t make them understand in detail.” (2, Sylheti, M, 61) “I don’t know English and they are helping, it’s a big matter. They are respecting that I came from Bangladesh, and I know English and they are helping me. They are not letting me understand that I don’t know. I don’t know it’s not a matter, they are helping me. They are making me understand that there is nothing to fear when I go to the doctor’s as I don’t know English, they will help me. It feels good.” (145, Sylheti, F, 35) “I like that they give me an interpreter. It helps me. I get the medicine, I am able to speak properly, I am not scared of anything like saying something wrong or being taking the wrong medicine.” (415, Hindi, F, 60) “I feel hesitant about how to speak, there is a third person who will know about my health issue. But as I cannot speak English, I have to tell them. Otherwise, I won’t get proper treatment or won’t get medicines. For this I have to tell them.” (154, Sylheti, F, 65)
Preference for face-to-face interpreting	“[t]he problems can be broken down and explained in a face-to-face interpretation. It cannot be done over the phone… over the phone, they talk in English at first and then they translate it in Bengali, you know. Therefore, it is hard to understand. If it is a face-to-face conversation, I can easily explain my problem in a face-to-face conversation, one can clearly explain one’s problem, and it is easier to express what that person feels.” (134, Sylheti, M, 47). “[s]ometimes when I am speaking, the interpreter’s connection crashes, and then he is not available anymore. Then it is said that one has left, then another one is brought, it happens a lot.” (408, Sylheti, M, 54).

### Patient engagement focus groups

We discussed our findings with Bangladeshi and Somali members of the public (*n* = 28). Their responses presented four main ideas, which resonated strongly with our research findings and helped develop our implications for practice (Table 5) [22]. The first covered the role played by GP reception staff in raising awareness and signposting of GP interpreting services. The second was related to concerns about the quality, accessibility and availability of services, where participants discussed the issue of community languages with multiple dialects and how online booking systems could impede access to healthcare. The third concerned professional interpreters who were deemed important when discussing private health matters that could not be disclosed to family members. Finally, there was discussion about how family members provide a valued and trusted source of emotional/communicative support. Yet where GPs excluded family members from consultations (e.g. privacy concerns during sexual health discussions), the reasons were not always explained, leading to patient confusion.

**Table 5:** Implications for practice based on existing commissioning guidance [33]

Principle	Summary of principle	Key recommendations	Insights/actions from current study
1.Access to services	Patients should be able to access primary care services in a way that ensures their language and communication requirements do not prevent them receiving the same quality of healthcare as others.	-Services are free at point of delivery -Services are high quality and accessible -Patients should not be asked to bring their own interpreter -Provide additional time (e.g. double appointments) -Record language/ communication preferences in patient’s records	-Patients to be provided with accessible information about the availability of professional services to raise awareness, alongside improving patient confidence in quality of professional services. -Emphasise the difference between professional services and other types of language support (e.g. family members). -Reassure patients that their family can come even when a professional interpreter is present. -Highlight challenges and risks associated with relying on informal language support. -Support practices to identify language needs and get this right from the first time a patient is registered. -National guidance to support consistent recording of interpreting use in electronic health records.
2. Booking interpreters	Staff working in primary care provider services should be aware of how to book interpreters across all languages, including BSL, and book them when required.	-Primary care provider is responsible -Should provide name and gender of interpreter -Interpreters should be regulated -Staff training	-Staff to offer professional interpreters proactively and on a regular basis. -Practice to mitigate inaccessibility of online booking systems for patients with language needs.
3. Timeliness of access	Patients requiring an interpreter should not be disadvantaged in terms of the timeliness of their access.	-Raise awareness (e.g. on registration and through advertising services) -Prioritise timeliness	-Support practices to raise awareness of professional interpreting services (e.g. through electronic screens in waiting rooms, community events). -Resource allocation formulas should take language need into account.
4. Personalised approach	Patients should expect a personalised approach to their language and communication requirements recognising that “one size does not fit all.”	Account for: -Preferences (e.g. gender, cultural identity) -Choice of modality -Limits of other frontline staff skills to assist patients	-Where possible, patients should be given a choice in the type of language support they are offered (e.g. face-to-face/telephone). -Provide practical steps for clinicians/interpreters to improve communication within the encounter [34]. For example, use the teach-back method to check understanding.
5. Opportunities to express views (i.e. compliments, comments, concerns and complaints)	Patients and clinicians should be able to express their views about the quality of the interpreting service they have received, in their first or preferred language and formats (written, spoken, signed etc.)	-Enable feedback opportunities -Produce service satisfaction reports -Ongoing monitoring	-Use existing survey tools to capture patient experience [10]. -Optimise mechanisms for patients/clinicians to provide feedback on the quality of interpreting services and in various formats (e.g. not relying on written feedback).

## Discussion

By focusing on participant experiences, this study provides new insight into individual influences on uptake of professional interpreting service in general practice and helps us to move beyond “the language problem” [23] to understand how people’s experiences and attitudes impact their decisions to engage with services (or not). We found that while most participants had previously been asked about their language support needs by their GP practice, they often had to advocate for language support. This was particularly relevant when requesting an appointment, where the move to online booking systems introduced additional challenges. Participants chose different types of language support due to the perceived benefits of involving family and friends, as well as their own appraisal of the seriousness of the presenting symptom/issue. Lack of confidence in professional interpreting services in GP practices resulted in examples of participants choosing alternative solutions (e.g. home remedies or seeking help elsewhere). The modality of interpreting was important to participants, who overall described a preference for face-to-face interpreting. Mirroring findings from a large-scale national survey [10] from which this sub-sample is drawn, translation apps (e.g. Google Translate) were discussed infrequently.

Challenges with service impermeability are well described in the literature [24] and our results support growing concerns about how remote (e.g. telephone interpreting) and digital systems (e.g. online booking tools) exacerbate access issues by creating another layer that requires family/ friends’ support [25]. These issues are more pronounced for people with limited English proficiency because of the assumptions made about people using services, the inflexibility of systems in supporting articulation and the role of others (e.g. family) in digitally representing the person as well as the nature, seriousness and course of presenting issue. The concept of “digital precarity” captures the experience of accessing a digitalised healthcare service, and emphasises intersecting influences on access, including age, migration status, gender and socioeconomic status [26]. A previous study focused on understanding South Asian and Middle Eastern patients’ perspectives on medicine-related problems emphasised the role of family and friends in adjudicating for medicine use (e.g. providing written and spoken translation) [27]. Although in our study we found GP practices sometimes discouraged friends and families as interpreters, the use of informal interpreters was commonplace. Despite concerns about confidentiality and the disclosure of certain health conditions, patients supported their use and showed a general lack of awareness of the potential implications of involving family and friends both for the patient themselves (e.g. accuracy, safeguarding) and the other party (e.g. hearing bad news first). The inadvertent negative impact of relying on informal interpreters has been previously highlighted [26, 28] and it will be important in future guidance to highlight the different roles that professional interpreters and family and friends play. For example, by emphasising the difference between professional services and other types of language support (e.g. family members) and reassuring patients that their family can come even when a professional interpreter is present.

Preferences for language support depended on participants’ appraisal of their symptoms or presenting problem, with people willing to “get by” without interpreting when issues were perceived as less serious. This may result in harm, as psychological research has shown that people are not always able to accurately appraise their symptoms [29]. This needs to be accounted for when promoting services to patients.

We also identified that coding of language need in the medical records could help practice staff know when to book interpreters, but staff may need additional support or training to ensure offers of interpreting services are made. Poor coding of language needs has been previously noted. For example, a US hospital-based study reported that, although electronic records included a language field, these data were only accurately populated 30% of the time, precluding the identification of patients with limited English language proficiency [30].

Participants described significant challenges with access and perceived variation of service quality. These beliefs could impact whether and how people accessed care for their health problems. Alhomoud et al. (2015) also showed that when patients were unable to access a professional interpreter, they managed their own care and made decisions without consulting other sources [27]. An interview study with asylum seekers and refugees reported that patients who could not access interpreters would avoid seeking help in primary care [28]. However, our study participants also reported emotional reassurance from engaging with professional interpreting services, emphasising “there is nothing to fear.” These positive accounts could be useful to build engagement in those who may have given up on services.

Participant preference for face-to-face interpreting is mirrored in the literature with healthcare professionals. For example, in one study, primary care professionals also described their reticence in using interpreting in telephone consultations due to concerns about confidentiality and the ability to detect cues [31] and practical challenges including long waits and mobile phone signal problems [32]. Limited availability of face-to-face interpreting, alongside poor-quality and hard to access telephone interpreting led some clinicians to use risky workarounds (e.g. Google Translate) [32].

### Implications for practice

Table 5 summarises how the themes can amplify existing guidance for commissioning and delivering professional interpreting services [33]. These have been organised according to five (of eight) key principles related to providing equitable access to services, awareness of booking interpreters, ensuring timeliness of access, providing a personalised approach and providing opportunities for patient and clinician feedback on services. These additional insights, from our findings and our public and patient engagement work, help operationalise the current guidance, by giving concrete examples of how to ensure their implementation. However, some of these actions are likely to be more feasible to implement in practice (e.g. raising awareness of services) than others (e.g. changing attitudes towards using friends/ family as interpreters; consistent recording of language need in electronic records). Given the pressures on general practice, this also includes a recognition that resource allocation formulas should take language provision need into account. Adopting these actions in general practice is essential to ensuring equitable healthcare.

### Strengths and limitations

Our study focused on understanding individual influences on uptake of professional services. However, implementation theory, such as the Consolidated Framework for Implementation Research (CFIR) [35] acknowledges that there are multiple spheres of influence not necessarily captured by focusing on participants’ experiences. Optimising uptake and experience of services will require work across domains of influence, including individual (lived experience), inner setting (context such as GP practice) and outer setting (health policies/ laws). Other examples of areas that need attention to ensure effective implementation, include establishing how interpreting services are monitored/evaluated and how technological infrastructure within GP practices is made compatible with services [36]. Conducting multilingual research presents unique challenges. For example, our participants were not recruited and interviewed by the research team which could limit our analysis; however, this work was carried out by linguistically and culturally-concordant, trained researchers. We worked closely with the researchers to ensure the interviews were conducted in line with our robust methodological approach and mitigate potential inconsistencies in quality. Difficulties recruiting participants from Indian and Pakistani backgrounds within certain age, sex, and region categories led to an oversampling of participants from Bangladeshi backgrounds. Our study focusses on one specific service area (primary care), and the broader applicability to other stages of healthcare may vary. While we concentrated on participants from South Asian backgrounds, through our community engagement work, our findings resonated strongly with people from Somali backgrounds and therefore are likely to be translatable to other ethnic groups.

## Conclusions

Participants from South Asian backgrounds with no/limited English proficiency face several challenges when accessing primary care, often needing to advocate for language support themselves. Several participants reported that they had been directed to use online booking tools to make appointments. Patients’ unmet language needs often led to the need to involve family members during the booking process. Family members were seen as valued and trusted sources of support, however it remains vital that the benefits of using professional trained interpreters are relayed to patients, for example to avoid situations where patients forgo using professional interpreters for matters self-assessed as less serious. Whilst participants suggested the quality of language support could vary between consultations, there was a clear preference for face-to-face interpreting.

## Electronic supplementary material

Below is the link to the electronic supplementary material.

Supplementary Material 1

Supplementary Material 2

## Data Availability

The datasets generated and/or analysed during the current study are not publicly available because study participants who participated did not consent to this. However, consent was obtained to share de-identified data with other researchers, which will be made available from the corresponding author on reasonable request.

## Footnotes

1 An interpreter who is qualified and registered with a relevant regulator, as per UK guidance for interpreting in primary care.
