# Supplementary material for: South Asian patient experiences of professional interpreting service provision in general practice in England: a qualitative interview study
Source: Int J Equity Health. 2025 Apr 16;24:104. doi: 10.1186/s12939-025-02477-4 (PMC12004599; doi:10.1186/s12939-025-02477-4)
Supplement: Supplementary file 1 — Supplementary Material 1 [file 12939_2025_2477_MOESM1_ESM.docx]

Supplementary material

1. Interview topic guide
2. Interview topic guide

| [Introduce yourself – check for questions based on PIS – check confidentiality understanding – take formal consent-check about audio recording]   - *Trying to understand people’s experiences of accessing healthcare from their GP surgery and using different types of language support (professional interpreter: in person/telephone/video, informal interpreter)* - *Need to know how the consultation goes (and how the different roles worked within the consultation)* - *Interested to hear how your experiences of different types of service compare and if you haven’t used* an interpreter provided by your GP surgery*, why that might be.* | |
| --- | --- |
| For those **reporting use of an interpreter provided by the GP surgery within the last six months** Aim to recruit n=20 people reporting using a professional interpreter | For those **not reporting recent use of an interpreter provided by their GP surgery but have been to their GP surgery and used an informal interpreter**  Aim to recruit n=10 people not reporting use of a professional interpreter, but NOT those reporting that their HCP speaks their language. We want to understand about different types of informal interpreting and how it compares. |
| In the survey, you told us that you had recently seen a healthcare professional from your GP surgery, and used the interpreting services **provided** by your GP surgery. In this interview, I would like to ask you some questions about the **most recent** time you used **an interpreter provided by your GP surgery**.  1. Tell me the story of how you last came to see a healthcare professional at your GP surgery and used an interpreter provided by the GP surgery.   - When did this last happen?   *• Please tell me all the experiences and the events which were important*  *• Start wherever you like*  *• Please take the time you need*  *• I’ll listen first, I won’t interrupt*  *• I’ll just take some notes in case I have any further questions for after you’ve finished telling me about it all* | In the survey, you told us that you had recently seen a healthcare professional from your GP surgery, but you **did not** use the interpreting services provided by the GP surgery. For instance, a family member or friend, health advocate, or other bilingual practice staff member may have acted as an **informal interpreter** for you; or you might have used a translation app such as Google Translate. In this interview, I would like to ask you some questions about the **most recent** time you saw a healthcare professional from your GP surgery but **did not** use an interpreter provided by the GP surgery i.e., you used an informal interpreter?  1. Tell me the story of how you last came to see came to see a healthcare professional at your GP surgery and did not use an interpreter provided by the GP surgery.   - When did this last happen?   *• Please tell me all the experiences and the events which were important*  *• Start wherever you like*  *• Please take the time you need*  *• I’ll listen first, I won’t interrupt*  *• I’ll just take some notes in case I have any further questions for after you’ve finished telling me about it all* |
| 2. Can you tell us how you made the decision to use the interpreting services provided by your GP surgery for your most recent consultation?   - When did this last happen? - Could you describe the person who interpreted for you e.g., their sex, their approximate age, or anything else you noticed about them? - Can you tell me about how the interpreter was arranged for you by the GP surgery? - Do you always use the interpreting services provided by your GP surgery, or do you sometimes use a different approach, can you explain? | 2. Can you tell us a bit about why you didn’t use the interpreting services provided by your GP surgery for your most recent consultation?   - Who interpreted for you? - Could you describe the person who interpreted for you e.g, their sex, their approximate age? - How did you decide who would interpret for you during this appointment? - Was there a specific reason you wanted to use this person as the interpreter? - Can you tell me about how you arranged for this person to interpret for you? - Do you always use this person, or do you sometimes use a different approach, can you explain? |
| 3. What was your experience like using the interpreter provided by your GP surgery?   - Could you describe how the interpreting worked in your most recent appointment? For instance was the interpreter with you at the GP surgery, or did they interpret over the telephone or by video call? | 3. What was your experience like with the informal interpreter/when this person interpreted for you?   - Could you describe how the interpreting worked in your most recent appointment? For instance, was [the informal interpreter/this person] with you at the GP surgery, or did they interpret over the telephone or by video call? |
| If telephone/video was used ask:  4a. How did the consultation work having the interpreter there?  4b. How did the technology work? For instance, how well could you see/ hear the interpreter? | If telephone/video was used ask:  4a. How did the consultation work having [the informal interpreter/this person] there?  4b. How did the technology work? For instance, how well could you see/hear [the informal interpreter/this person]? |
| 5. How did the conversation and interaction work between yourself, the interpreter and the healthcare professional?   - For instance, how well did the conversation flow? - How well were you able to express yourself to get across what you wanted to say? - If relevant, to what extent were you able to demonstrate or highlight any health concerns via visual cues such as pointing? - What impact, if any, did any technology used during your most recent appointment (e.g., telephone/video) have on the conversation or interaction? | 5. How did the conversation and interaction work between yourself, [the informal interpreter/this person] and the healthcare professional?   - For instance, how well did the conversation flow? - How well were you able to express yourself to get across what you wanted to say? - If relevant, to what extent were you able to demonstrate or highlight any health concerns via visual cuees such as pointing? - What impact, if any, did any technology used during your most recent appointment (e.g., telephone/video) have on the conversation or interaction? |
| 6. How did it feel having an interpreter provided by your GP surgery there?   - How easily were you able to understand what was happening? - How easily could you make yourself understood e.g., to express your views, concerns, needs, emotions to the healthcare professional through the interpreting service? - Did you feel the healthcare professional was speaking to you or the interpreter? | 6. How did it feel having [the informal interpreter/this person] there?   - How easily were you able to understand what was happening? - How easily could you make yourself understood e.g., to express your views, concerns, needs, emotions to the healthcare professional through the interpreter? - Did you feel the healthcare professional was speaking to you or the interpreter? |
| 7. To what extent did you feel able to talk freely in the consultation?   - Did you have any concerns e.g., about confidentiality? | 7. To what extent did you feel able to talk freely in the consultation?   - Did you have any concerns e.g., about confidentiality? |
| 8. To what extent did you feel that the healthcare professional understood your problems?   - If not, why do you think this was? | 8. To what extent did you feel that the healthcare professional understood your problems?   - If not, why do you think this was? |
| 9. Would you use an interpreter provided by your GP surgery for different health concerns/appointments?   - Are there any health concerns you wouldn’t feel comfortable using this type of service for, if so, why? | 9. Would you use this [informal interpreter/person] for different health concerns/appointments?   - Are there any health concerns you wouldn’t feel comfortable using this [informal interpreter/ person] for, if so why? |
| 10. Was this the first time you used the an interpreter provided by your GP surgery? If not, how did your most recent experience compare with previous experiences? | 10. Was this the first time you used [the informal interpreter/this person]? If not, how did your most recent experience compare with previous experiences? |
| 11. Can you tell me what you liked about using using the interpreter provided by your GP surgery, and also what you did not like about it? | 11. Can you tell me what you liked about using [the informal interpreter/this person], and also what you did not like? |
| 12. Do you have other experience of language support, in other words not using an interpreter provided by your GP surgery?   - If, so, how do the different experiences compare? - Which form of language support do you prefer using and why? | 12. Do you have other experience of language support, such as using an interpreter provided by your GP surgery)?   - If so, how do the different experiences compare? - Which form of language support do you prefer using and why? |
| 13. Can you tell us about the impact of COVID-19 on how you access healthcare at your GP surgery?   - To what extent have the language support options available to you at your GP surgery changed since the pandemic? | 13. Can you tell us about the impact of COVID-19 on how you access healthcare at your GP surgery?   - To what extent have the language support options available to you at your GP surgery changed since the pandemic? |
| 14. If you had not been able to access/use an interpreter provided by your GP surgery, what would you have done? | 14. If you had not been able to access/use [this informal interpreter/person/type of language support], what would you have done? |
| 15. What kind of suggestions would you make for improving the interpreting services provided by your GP practice?   - What would “ideal” look like? - How could remote interpreting services e.g., interpreting by telephone/video be improved. | 15. What kind of suggestions would you make for improving the interpreting services provided by your GP practice?   - What would “ideal” look like? - How could remote interpreting services e.g., interpreting by telephone/video be improved. |
| 16. Is there anything else you would like to tell us about interpreting services at your GP practice? | 16. Is there anything else you would like to tell us about interpreting services at your GP practice? |
