# Supplementary material for: South Asian patient experiences of professional interpreting service provision in general practice in England: a qualitative interview study
Source: Int J Equity Health. 2025 Apr 16;24:104. doi: 10.1186/s12939-025-02477-4 (PMC12004599; doi:10.1186/s12939-025-02477-4)
Supplement: Supplementary file 2 — Supplementary Material 2 [file 12939_2025_2477_MOESM2_ESM.docx]

Supplementary File 1: COREQ checklist

Consolidated criteria for reporting qualitative studies (COREQ): 32-item checklist

Developed from:

Tong A, Sainsbury P, Craig J. Consolidated criteria for reporting qualitative research (COREQ): a 32-item checklist for interviews and focus groups. International Journal for Quality in Health Care. 2007. Volume 19, Number 6: pp. 349 – 357

| **Item No** | | **Guide Questions/Description** | **Reported on Page #** |  |
| --- | --- | --- | --- | --- |
| **Domain 1: Research team and reflexivity** | | | |  |
| **Personal Characteristics** | | | |  |
| 1. Interviewer/ facilitator | | Which author/s conducted the interview or focus group? | Pg. 7 |  |
| 2. Credentials | | What were the researcher’s credentials? E.g., PhD, MD | Pg. 1 |  |
| 3. Occupation | | What was their occupation at the time of the study? | Pg. 1 |  |
| 4. Gender | | Was the researcher male or female? | Mixed |  |
| 5. Experience and training | | What experience or training did the researcher have? | The sub-contractor used trained community researchers to conduct the interviews. Briefing sessions were held between the sub-contractor and the academic research team prior to the start of the interview study. Each community researcher then attended a briefing session led by the sub-contractor, where guidance on the conduct of the interviews was issued.  The academic research team included two research fellows, one reader, four professors, as well as the study’s PPIE lead who is also an interpreter. |  |
| **Relationship with participants** | | | |  |
| 6. Relationship established | | Was a relationship established prior to study commencement? | N/A |  |
| 7. Participant knowledge of the interviewer | | What did the participants know about the researcher? e.g. personal goals, reasons for doing the research? | Pg. 7 |  |
| 8. Interviewer characteristics | | What characteristics were reported about the interviewer/facilitator? e.g. Bias, assumptions, reasons and interests in the research topic | The community researchers who conducted the interviews spoke the same languages as the research participants.  The academic research team members have diverse backgrounds e.g., social sciences, psychology, health inequalities, and translation and interpreting studies. The PPIE lead for the study is a co-author of this article. |  |
| **Domain 2: study design** | | |  |  |
| **Theoretical framework** | | |  |  |
| 9. Methodological orientation and Theory | What methodological orientation was stated to underpin the study? e.g. grounded theory, discourse analysis, ethnography, phenomenology, content analysis | Pg. 7-8 |  |  |
| **Participant selection** | | |  |  |
| 10. Sampling | How were participants selected? e.g., purposive, convenience, consecutive, snowball | Pg. 7 |  |  |
| 11. Method of approach | How were participants approached? e.g., face-to-face, telephone, mail, email | Pg. 6-7 |  |  |
| 12. Sample size | How many participants were in the study? | Pg. 6-7, 8 |  |  |
| 13. Non-participation Setting | How many people refused to participate or dropped out? Reasons? | Pg. 7 |  |  |
| 14. Setting of data collection | Where was the data collected? e.g., home, clinic, workplace | N/A |  |  |
| 15. Presence of nonparticipants | Was anyone else present besides the participants and researchers? | N/A |  |  |
| 16. Description of sample | What are the important characteristics of the sample? e.g. demographic data, date | Pg. 8 (table 1) |  |  |
| **Data collection** | | |  | No |
| 17. Interview guide | Were questions, prompts, and guides provided by the authors? Was it pilot tested? |  |  |  |
| 18. Repeat interviews | Were repeat interviews carried out? If yes, how many? | N/A |  |  |
| 19. Audio/visual recording | Did the research use audio or visual recording to collect the data? | Pg. 7 |  |  |
| 20. Field notes | Were field notes made during and/or after the interview or focus group? | N/A |  |  |
| 21. Duration | What was the duration of the interviews or focus group? | Pg. 7 |  |  |
| 22. Data saturation | Was data saturation discussed? | N/A |  |  |
| 23. Transcripts returned | Were transcripts returned to participants for comment and/or correction? | N/A |  |  |
| **Domain 3: analysis and findings** | | |  |  |
| **Data analysis** | | |  |  |
| 24. Number of data coders | How many data coders coded the data? | Pg. 7-8 |  |  |
| 25. Description of the coding tree | Did the authors provide a description of the coding tree? | N/A |  |  |
| 26. Derivation of themes | Were themes identified in advance or derived from the data? | Pg. 4-5 |  |  |
| 27. Software | What software, if applicable, was used to manage the data? | Pg. 7 |  |  |
| 28. Participant checking | Did participants provide feedback on the findings? | N/A |  |  |
| **Reporting** | | |  |  |
| 29. Quotations presented | Were participant quotations presented to illustrate the themes/findings? Was each quotation identified? e.g., participant number | Yes, participant quotations are presented in the findings section. Each quotation is identified by a participant ID number. |  |  |
| 30. Data and findings consistent | Was there consistency between the data presented and the findings? | Yes, see Findings and supporting tables (Tables 2-4). |  |  |
| 31. Clarity of major themes | Were major themes clearly presented in the findings? | Yes, see Findings and supporting tables (Tables 2-4). |  |  |
| 32. Clarity of minor themes | Is there a description of diverse cases or a discussion of minor themes? | See pg. 7-8 for description of analytical approach |  |  |
